# Ionic Liquid Vapors in Vacuum: Possibility to Derive Anodic Stabilities from DFT and UPS

**DOI:** 10.1021/acsomega.0c05369

**Published:** 2021-02-15

**Authors:** Ivar Kuusik, Mati Kook, Rainer Pärna, Vambola Kisand

**Affiliations:** Institute of Physics, University of Tartu, W. Ostwaldi 1, EE-50411 Tartu, Estonia

## Abstract

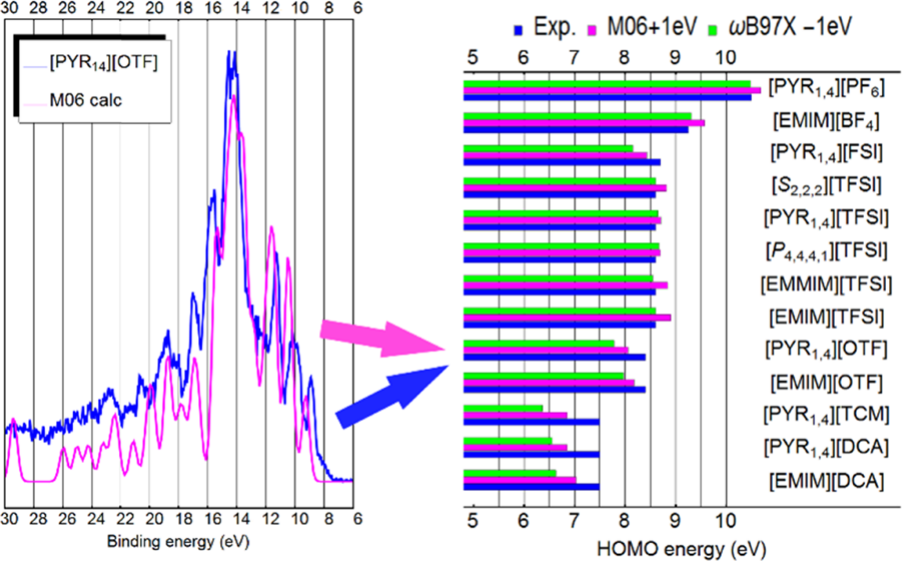

Ultraviolet photoelectron spectroscopy (UPS) investigations of several gas-phase ionic liquid (IL) ion pairs have been conducted. [EMIM][OTF], [PYR_14_][OTF], [EMIM][DCA], [PYR_14_][DCA], [PYR_14_][TCM], [PYR_14_][FSI], [PYR_14_][PF_6_], [S_222_][TFSI], [P_4441_][TFSI], and [EMMIM][TFSI] vapor UPS spectra are presented for the first time. The experimental low-binding-energy cutoff value (highest occupied molecular orbital, HOMO energy) of the ionic liquid ion pairs, which is of great interest, has been measured. Many studies use calculated gas-phase electronic properties to estimate the liquid-phase electrochemical stability. Hybrid density functional theory (DFT) calculations have been used to interpret the experimental data. The gas-phase photoelectron spectra in conjunction with the theoretical calculations are able to verify most HOMO energies and assign them to the cation or anion. The hybrid M06 functional is shown to offer a very good description of the ionic liquid electronic structure. In some cases, the excellent agreement between the UPS spectra and the M06 calculation validates the conformer found and constitutes as a first indirect experimental determination of ionic liquid ion-pair structure. Comparisons with recent theoretical studies are made, and implications for electrochemical applications are discussed. The new data provide a much-needed reference for future ab initio calculations and support the argument that modeling of IL cations and anions separately is incorrect.

## 1 Introduction

Ionic liquids (ILs) are generally defined as molten organic salts with a melting point below 100 °C. ILs have attracted interest because of their uncommon physicochemical properties such as low melting temperatures, excellent solvation ability, relatively high thermal stability, low vapor pressure, nonflammability, high electrochemical stability, etc.

One important application for ionic liquids is as an electrolyte in electrochemical double-layer capacitors (EDLCs) or supercapacitors. In that application, the ionic conductivity and the width of the electrochemical stability window (EW) are the most important properties. Although generally the viscosity of ILs is higher and the ion conductivity is lower than in the conventional electrolytes, ILs are nonetheless considered as the ideal working electrolytes for EDLCs because of their large electrochemical windows, excellent thermal stability, and negligible volatility.^1^ In supercapacitors, ILs are close to commercial viability.^2^

ILs are also very promising for use in Li batteries, as many ILs are intrinsically stable against the solid Li anode and some ILs form stable solid electrolyte interface layers, thus inhibiting the dendrite growth problem that plagues many Li-battery designs. The wide EW of ILs is of critical importance^3^ and allows the use of high cathode voltages and enables the design of high-voltage batteries. ILs may also open up alternative battery chemistries in the standard Li-ion batteries, e.g., Li-metal or metal-air.^2^

It is possible to synthesize a vast number of different ILs as there are a large number of different cations (and anions) available. Each class of cations for ILs has advantages and disadvantages. Imidazolium- and pyrrolidinium-based ILs, in particular, are very promising for EDLCs and Li batteries. The latter have a more charge-localized aliphatic structure and also a higher EW than the delocalized imidazolium-type aromatic cations.^4^ Ammonium- and pyrrolidinium-based ILs have outstanding electrochemical stability because these saturated heterocyclic cations have superior resistance toward reduction.^5^ Sulfonium- and phosphonium-based cations should also be of interest as they have a high ionization potential and should therefore also be very stable electrochemically.

The electronic structure of a diverse set of ILs was investigated in this study. We focused on the question of how to determine the intrinsic EWs of the ILs and their oxidation stabilities. The intrinsic (oxidation) stability is the electrolyte stability without interaction with the electrode surface or specific interaction, such as hydrogen bonding with other electrolyte components.^6^ Ion transfer is neglected, and the electrode is considered to be chemically inert. In this approach, the electrode in contact with the IL is considered as “electron reservoir” and only electron transfer between electrode and the IL is considered. Only when there are no ion dissociation or chemical reactions occurring, this one-electron redox mechanism becomes the limiting factor of the anodic and cathodic stabilities.^6,7^

There are numerous (recent) studies on this topic, and there is a wide range of EW values and different orders of anion stabilities given in the literature.^3,4,6,8−15^ A short overview of some common EW calculation methods is presented before the new data are analyzed.

In the simplest approximation, the electrochemical stability window (EW) of ILs is determined by the highest occupied molecular orbital (HOMO) and lowest unoccupied molecular orbital (LUMO) energies of the ion pairs they consist of
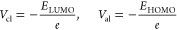
1This so-called frontier molecular orbital method was already proposed by Ong et al.^12^ and has been used actively ever since.^8^ Ilawe et al. also claimed that the HOMO–LUMO gap of the ion pairs is an indicator of IL stability.^10^

Asha et al. suggested that the critical factor that decides the EW of pyrrolidinium-based ILs is the HOMO energy of pairing anions.^4^ Some studies go even further and suggest that the electrochemical window of ionic liquids can be estimated by the oxidation and reduction potentials of the constituent ions (in vacuum or in some solvation models)^3^
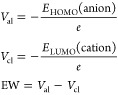
2The next approximation to the EW was to take the common electronic gap of the cations and anions (i.e., the overlap). For example, Ong et al. and Lian et al. claimed that the cathodic and anodic limits can be reasonably estimated from the relation
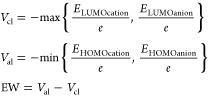
3Here, the energies of the individual cations and anions (in vacuum) are used.^1,12^ Using eq 3, Asha et al. found an agreement with the experimental EW values with a maximum deviation of 5.52% and a minimum deviation of 1.19%.^4^ Lian et al. even claimed that this calculation procedure has already been validated.^9^

The thermodynamic method has also been used for predicting the liquid-phase EWs. However, in practice, it is also based on the calculation of oxidation and reduction energies of free ions and their solvation energies. Liquid environments can be approximated by calculating the ions in effective mediums (polarizable continuum model, PCM approximation or other solvation models) or by generating molecular dynamics (MD) equilibrated snapshots as input for density functional theory (DFT) calculations.

Computational modeling of ILs requires great care. ILs have been described as “room temperature plasma” due to their charged internal structure with highly correlated motion. For example, most of the currently widely used DFT functionals do not describe anions adequately due to the incorrect behavior of the long-range interactions, while classical force fields used for molecular dynamics (MD) simulations do not readily deal with the partial charge transfer between anions and cations of an IL.^2^

All of these HOMO and LUMO energy-based EW estimations (eqs 1–3) rely on the so-called ion-pair approximation. The ion-pair approximation assumes that the electronic structure of the ionic liquid is similar to the ion pairs it consists of. The validity of the ion-pair approximation for ILs has experimentally only been studied in a few cases. In [EMIM][TFSI], the gas- and liquid-phase electronic structures were indeed very similar and the ion-pair approximation seems to hold.^16−20^

In the case of [EMIM][BF_4_], the ion-pair approximation seems to hold overall, as the ion-pair calculation is able to describe the overall shape of the liquid-phase ultraviolet photoelectron spectroscopy (UPS) spectrum very well. However, the description of the top of the valence band of the liquid (or HOMO level of the ion pair) is not accurate using DFT and the ion-pair approximation.^21,22^ The situation is even worse in [EMIM][B(CN)_4_], where the ion-pair approximation is quantitatively unable to describe the liquid-phase electronic structure.^23^ Importantly however, when performing proper bulk calculations of these ILs, DFT is again able to qualitatively reproduce the correct electronic structure.^22,23^ These bulk calculations still lack quantitative accuracy and are computationally too expensive to perform using hybrid functionals. This explains the numerous studies on the liquid IL properties that have been performed using the ion-pair approximation.

There are still very limited experimental data about the gas-phase properties of IL vapors. The knowledge of the electronic energies and composition of the topmost valence states is vital for understanding of processes that involve the removal of electrons from the IL.^24^ Due to the high practical importance of the EW and the very large number of ab initio calculation studies of ionic liquid ion pairs in vacuum, further experimental data on the HOMO (and LUMO) states of ionic liquid ion pairs are needed.

Therefore, we have investigated the electronic structure of this diverse set of ILs with the emphasis on their intrinsic electrochemical stability.

The ILs under investigation in this work and their abbreviations are shown in Tables 1 and S1. We use the simplest notation for the ILs, which is based on the cations and anions: [CATION][ANION]. To the best of our knowledge, this is the first presentation of the vapor-phase UPS spectra of [EMIM][OTF], [PYR_14_][OTF], [EMIM][DCA], [PYR_14_][DCA] (supplementary), [PYR_14_][TCM], [PYR_14_][FSI] and [PYR_14_][PF_6_] (supplementary), [S_222_][TFSI], [P_4441_][TFSI] and [EMMIM][TFSI].

**Table 1:** ILs Investigated in This Work

ionic liquid abbreviation	possible thermal degradation (change in spectra)^a^	HOMO (exp.) (eV)	M06 HOMO −1 eV	ωB97X-D HOMO +1 eV	HOMO localization and limitation of the anodic potential
[S_222_][TFSI]	very little	8.6	–8.81	–8.60	ANION
[P_4441_][TFSI]	very little	8.6	–8.69	–8.67	ANION
[EMIM][TFSI]	very little	8.6	–8.9	–8.60	MIXED
[EMMIM][TFSI]	very little	8.6	–8.84	–8.55	MIXED
[EMIM][OTF]	somewhat	8.4	–8.18	–7.97	MIXED
[PYR_14_][OTF]	somewhat	8.4	–8.06	–7.78	ANION
[PYR_14_][DCA]^d^	likely	7.5	–6.85	–6.55	ANION
[EMIM][DCA]^c^	highly likely	7.5	–7.03	–6.63	ANION
[PYR_14_][TCM]	somewhat	7.5	–6.85	–6.36	ANION
[PYR_14_][TFSI]	very little	8.6	–8.71	–8.65	ANION
[PYR_14_][FSI]	highly likely	8.7	–8.43	–8.15	ANION
[EMIM][BF_4_]^b^	somewhat	9.25^b^	–9.57	–9.31	CATION
[PYR_14_][PF_6_]^d^	highly likely	10.5	–10.68	–10.48	MIXED

a The possible thermal degradation column is a subjective evaluation based on the number of thermal decomposition products identified in the TOF-MS and photoelectron spectra and the reproducibility and stability of the UPS spectra.

b The [EMIM][BF_4_] UPS spectrum from ref (21) has been used, and the HOMO level has been reinterpreted (see the discussion below).

c The [EMIM][DCA] UPS spectrum is of low quality.

d The UPS spectrum is shown in the Supporting Information.

Our assumption is that the intrinsic (maximum) anodic limit determined from the ion-pair approximation may give some insights when comparisons between different ILs are made.

## 2 Experimental Section

The ionic liquids under study were purchased from different manufacturers. Their stated purities are shown in Table 1. Most ILs were transparent, but some were yellowish ([PYR_14_][DCA]).

The UPS measurements of the ILs were carried out at the new FinEstBeAMS beamline of the new MAX-IV 1.5 GeV storage ring (Lund, Sweden). The beamline is equipped with a collimating grazing incidence plane grating monochromator and toroidal focusing mirrors and covers the excitation photon energy range from 4.5 eV to about 1300 eV.^25^

The ionic liquids were evaporated from a quartz crucible in an effusion cell (MBE Komponeten). After inserting the IL into the effusion cell, heating of the IL at around 80 °C for several hours was performed to remove residual water from the chemical.

UPS measurements were carried out in the 10^–7^ mbar pressure range with a liquid-nitrogen-cooled cold trap.

**Figure 1:**
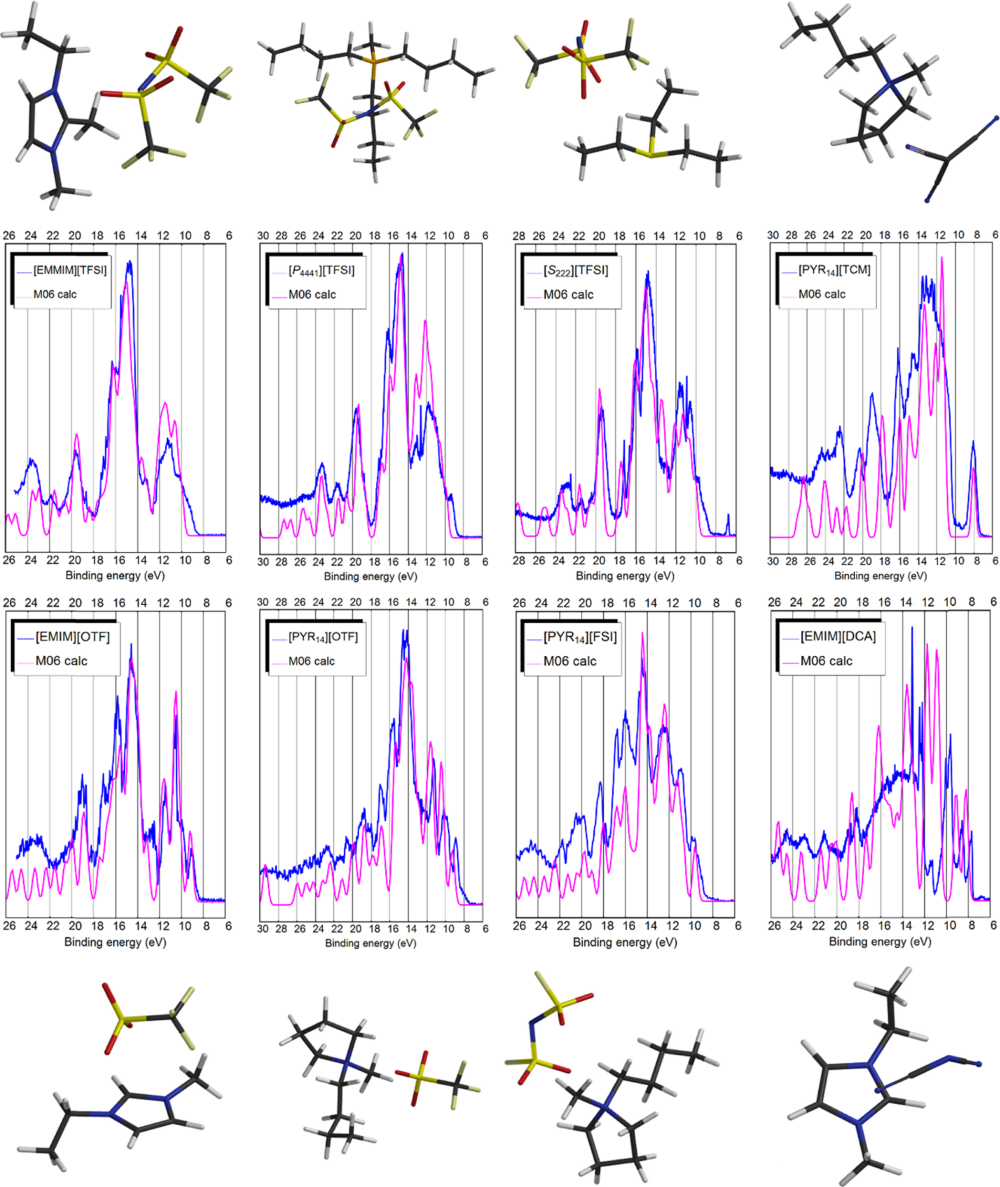
Predicted structures, experimental UPS spectra (blue curves), and DFT density of states (DOS) simulations (magenta curve) of IL ion pairs. The predicted ion structures are shown above the first row of UPS spectra and below the second row of the UPS spectra. The different bonds are shown using different colors: gray (hydrogen), black (carbon), blue (nitrogen), red (oxygen), green (fluorine), orange (phosphorus), and yellow (sulfur). The simulated spectra are based on the molecular structure shown.

The spectra were obtained using an electron energy analyzer (Scienta R-4000) in the fixed analyzer transmission mode. The measurements were performed with an excitation energy 40–50 eV and by using a spectrometer pass energy of 20 or 50 eV. Binding energies were calibrated to the H_2_O 1b_1_ (12.62 eV) photoelectron line. Due to the high hydrophilicity of some of the ILs and their high water content, water was still evaporating even at elevated temperatures, and traces of water vapor are visible in most spectra. Water contributes the peaks at 12.62 and 13.0 eV. If the water vapor signal was too strong, a molecular H_2_O spectrum was subtracted from the spectra shown in Figure 1 ([EMIM][OTF], [PYR_14_][FSI], [PYR_14_][OTF], [PYR_14_][DCA], [EMIM][DCA]). Due to the relatively low vapor density of the ionic liquid, some background gases also appear in the spectra, most notably nitrogen, which contributes peaks at 15.6, 16.7, 16.9, 17.15, and 18.75 eV. If the nitrogen signal was too strong, a molecular N_2_ spectrum was subtracted from the spectra shown in Figure 1 ([PYR_14_][DCA], [PYR_14_][TCM], [EMIM][OTF], [EMIM][DCA]). In most cases, the peaks from the background gases do not significantly influence the interpretation of the spectra.

At elevated temperatures, hydrogen fluoride (HF) photolines were seen at binding energies of 16.06 and 16.42 eV in the UPS spectra of [PYR_14_][FSI] and [PYR_14_][PF_6_].^26^ These distinct peaks were subtracted from the spectra. In addition, contributions from SO_2_ and CO_2_ were seen in the [PYR_14_][FSI] UPS spectra. Some SO_2_ features and the three sharp CO_2_ photolines at 13.78, 18.08, and 19.40 eV^27^ were subtracted from the [PYR_14_][FSI] spectrum. In the case of the ILs based on the cyano-anions, relatively weak HCN lines at 13.60–14.23 eV^27,28^ were seen in [PYR_14_][DCA] spectra, and these lines were stronger in [EMIM][DCA]. All of the above-mentioned gases are due to the thermal decomposition of the IL. The raw spectra are shown in the Supporting Information (Figures S1 and S2).

The origin of the spurious peaks at binding energies of 6.8, 11.0, and 22.55 eV in the UPS spectrum of [S_222_][TFSI] is unknown at this time.

In the case of the [PYR_14_][OTF] spectrum, the tail that extends from the lowest-binding-energy peak in the UPS spectrum (at about 9.0 eV) is ignored (see Figure 1). Then, the [EMIM][OTF] and [PYR_14_][OTF] low-energy cutoff values are the same, which is expected.

DFT calculations were performed using Spartan 14 software. The hybrid functionals M06 (includes 27% exact Hartree–Fock exchange) and ωB97X-D (100% Hartree–Fock exchange for long-range electron–electron interactions) were used for DFT calculations. The Gaussian basis set 6-311++G** (basis set with d,p polarization and diffuse functions) was used throughout.

Many different ion-pair conformers were manually constructed. All geometries (conformers) were optimized (relaxed) for the lowest energy. For example, over 40 different conformers were studied for [S_222_][TFSI]. Similarly to Fogarty et al., the emphasis was to survey a wide range of cation–anion placements.^29^ The density of states (DOS)-type spectra shown in Figure 1 were obtained by convoluting the calculated discrete states with a Gaussian function (0.5–0.6 eV full width at half-maximum) under the assumption that the electron emission intensities from each orbital are equal. Zero point energy and vibronic effects are not taken into account in the calculations.

The structures shown in Figure 1 represent the conformers whose calculated electronic structure agrees best with the experimental UPS spectra. The simulated DOS’s of the predicted conformers are shown next to the experimental UPS spectra.

It is important to point out that neither the experimental nor the theoretical spectra may represent the lowest-energy conformer, since the evaporation temperature was about 500 K. Furthermore, it has been shown recently that the prediction of IL ion-pair structure is complicated and depends on the calculation method.^30^

In some cases, a significant dependence of the calculated DOS on the underlying structure of the ion pair (conformer) was found, unlike in the work of Reinmöller et al.^31^ For example, the simulated DOS of [P_4441_][TFSI], [S_222_][TFSI], and [PYR_14_][DCA] are relatively sensitive on the ion-pair structure, while not so in the case of [PYR_14_][OTF], [EMIM][DCA], and [EMIM][OTF].

The calculated DOS was shifted to align the peaks and make a comparison between the experimental spectra and the simulated DOS easier. A shift of 2.2 eV was used for the DOS’s calculated with the M06 functional, which are shown in Figure 1. The ωB97X-D functional needed just a 0.5 eV shift (not shown). Importantly, these shifts are constant from one ion pair to the next and are probably due to some systematic deficiencies in the functionals.

## 3 Results

The experimental UPS spectra along with the calculated DOS-type spectra are shown in Figure 1. The figure also shows the ion-pair conformer used for the calculation, i.e., the predicted ion-pair structure. The low-energy cutoff values (HOMO energies) are shown in Table 1. The energy scale of our measurements is very similar to that given by Strasser et al.^18^^20^ However, most other studies have energy scales that are shifted to much lower energies.^16,17,31−35^

Yoshimura et al. have published liquid UPS spectra of [BMIM][PF_6_] and [BMIM][TFSI].^36^ Ulbrich et al. and Kanai et al. have measured the [BMIM][OTF] UPS spectrum.^35,37^ The recent work of Fogarty et al. provided many new XPS and resonant Auger spectra of ILs.^24^ Compared to our gas-phase data, their liquid-phase spectra are much more broadened, as expected. There are still very few published photoelectron spectra of IL vapors at this point.

Within our experimental signal-to-noise ratio, the HOMO energies of the TFSI anion-based ILs [S_222_][TFSI] and [P_4441_][TFSI], as well as the [PYR_14_][TFSI], [DEME][TFSI], and [EMIM][TFSI] spectra from the previous study,^19^ are similar. Their low-energy cutoff values are about 8.6–8.7 eV.

### 3.1 Calculation Method

The choice of a calculation method for ionic liquid ion pairs is not trivial. What calculation method one should use to estimate these HOMO and LUMO energies from eqs 1–3? It is well known that the HOMO and LUMO energies depend on the DFT functional used for the calculation.

Asha et al. recommended the DFT functional M06-L with the basis set 6-311++G(d,p) as the best method for the calculation of EWs. An agreement with the experimental values with a maximum deviation of 5.52% and a minimum deviation of 1.19% was claimed.^4^ Borodin et al. recommended the hybrid M05-2X functional, as it is closest to the G4MP2 ref (6). Jónsson et al. found that the similar hybrid M06-2X functional had the smallest deviation from the ΔCBS ref (13). The nonhybrid M06-L was also evaluated in both studies, and it had the largest deviation from the reference. Ilawe et al. recommended the ωB97X-D functional.^10^ They found that the ωB97X-D functional is superior to M06-2X and B3LYP. Lian et al. also recommended this hybrid functional as it is quantitatively accurate for predicting the electronic properties of individual ions in vacuum.^9^ In our previous UPS study of TFSI anion-based ILs, the ωB97X-D functional was shown to perform very well, with only a 0.5 eV energy scale shift required. This is not surprising since the ωB97X-D functional is very close to the best functionals in the benchmark studies.^38^ Fu et al. showed that B3LYP was accurate in the prediction of the adiabatic ionization potentials of 160 structurally unrelated organic molecules in gas phase.^39^ Tian et al. claimed that PCM calculations are superior to their gas-phase calculations performed using the (similar) B2PLYP-D functional. However, we have shown previously that B3LYP is a poor functional for the description of the electronic structure of ILs.^19,23^

The hybrid DFT functionals are better than standard GGA DFT functionals for the description of the [EMIM][BF_4_] ion pair. In the case of [EMIM][BF_4_] ion pairs, only the MP2 calculation described the HOMO level correctly.^21^ Similarly, in the case of [EMIM][B(CN)_4_], again only the MP2 ion-pair approximation was able to describe the top of the valence band.^23^ Surprisingly, in the case of [EMIM][TFSI] and [PYR_14_][TFSI], the MP2-calculated DOS had a too big separation of the HOMO level from the other orbitals.^19^ Therefore, both hybrid DFT and MP2 methods should be considered and investigated.

The energies (HOMO, LUMO, orbital, total, etc.) from the DFT calculations are dependent on the functional and basis set. Unfortunately, this makes ab initio calculation of the absolute binding energies difficult, and in many cases, empirical corrections are needed. We have used shifting of the DOS by 2.2 eV and corrections to the calculated M06 HOMO energies by 1 eV. Unfortunately, the Koopmans theorem is not valid within the DFT formalism, so the Δ-SCF method can be used for an improved estimate of the ionization energy. This calculation shows that the Δ-SCF^40^ M06 ionization energy is larger than the HOMO energy by about 1.6 eV, thus leaving only an about 0.8 eV shift. The calculation was performed for conformers at 0 K, and the neglect of zero point and thermal (broadening) energy is another reason why the calculated binding energies are not directly comparable to the experimental data.

Next, some ILs will be discussed separately in more detail.

### 3.2 [EMIM][BF_4_]

The vapor-phase photoelectron spectrum of [EMIM][BF_4_] has already been studied.^21^ It was found that the HOMO energy is about 7.4 eV, which seems too low in the context of the present study. For example, [EMIM][DCA] has a HOMO energy of 7.5 eV (see Table 1). The HOMO energy of [EMIM][BF_4_] may need a reevaluation. Therefore, some discussion about the electronic structure of [EMIM][BF_4_] is necessary at this point.

[EMIM][BF_4_] is actually a surprisingly difficult case from a computational standpoint. For example, it is rather challenging to correctly assign the HOMO to the cation or anion and to correctly estimate the electronic gap.

Haskins et al. calculated the HOMO–LUMO gap of [EMIM][BF_4_] as 6.56 eV using the hybrid HSE06 functional.^11^ Then, using the same HSE06 functional, Yildirim et al. calculated a gap of 7.11–7.48 eV.^41^ Later, they calculated a gap of 6.72 eV, again using the HSE06 functional, which is known to provide relatively accurate band gaps.^42,12^ However, when more ion pairs were added to the calculation of Haskins et al. (simulating a bulk environment), the gap dropped substantially—by about 1.4–5.15 eV.^11^ Similarly, Yildirim et al. found that the bulk gap of [EMIM][BF_4_] shrank quite substantially when going from the isolated ion pair to the bulk phase, while the electronic gap of [PYR_14_][TFSI], for example, remained unchanged.^41,42^ From their single-point calculations, Yildirim et al. found that the hybrid functionals predict a more accurate picture of the ion pair and that both HOMO and LUMO states are dominated by the cation.^41^

We interpret this as a failure of DFT to correctly describe the HOMO of imidazolium-based (aromatic) ILs. The correct cation π-states emerge as the top of the valence band in bulk calculations and in some hybrid ion-pair calculations. This has already been discussed previously.^21−23^ The (self-consistent) GW calculations are able to conclusively solve this issue and confirm that the HOMO–LUMO gap of [EMIM][BF_4_] is larger than 9 eV.^43^

In their gas-phase study of [EMIM][BF_4_], Kuusik et al. showed that MP2 is able to describe only two out of the three distinct outer valence band peaks (peaks A–C).^21^ Thus, it seems reasonable to assign the peaks B and C (see ref (21)) to [EMIM][BF_4_] and the peak A (A1 and A2) should no longer be interpreted as the HOMO state of the ion pair. The peak A could be due to dimers, trimers, or other small clusters. It could also result from some thermal degradation products. The HOMO energy is now interpreted as the low-binding-energy tail of the peak B, which is at 9.25 eV. In addition, in the [EMIM][BF_4_] photo-fragmentation study by Kuusik et al., the dominant cation threshold (appearance) energy was found to have a similar value: 9.43 eV.^44^ Using UPS and IPS, Kanai et al. showed that [OMIM][BF_4_] has a gap of 9.1 eV.^45^ That IL is expected to have a very similar gap to [EMIM][BF_4_] and [BMIM][BF_4_].

Unfortunately, these unexplicable low-energy tails in the UPS spectra of ILs are rather common. For example, Krischok et al., Reinmöller et al., and Höfft et al. published the UPS spectra of [EMIM][TFSI], where the low-energy tail extended to almost 2 eV binding energies!^17,31,32^ Ulbricht et al. had similar low-energy tails in the spectra of [BMIM][TFSI] and [BMIM][BF_4_].

This reinterpretation of the HOMO energy of [EMIM][BF_4_] is validated by the overall agreement with our calculation trends. For example, the adjusted M06 (−1 eV) HOMO energy is at −9.57 eV, which is in good agreement with 9.25 eV. Similarly, the adjusted ωB97X-D (+1 eV) value for [EMIM][BF_4_] is at −9.3 eV, which is in excellent agreement. Our M06 calculation yields HOMO and LUMO binding energies of 8.57 and 1.67 eV, respectively. Exactly the same 6.90 eV gap value was found by Haskins et al. in their M06 calculation.

Since the HOMO (and LUMO) of [EMIM][BF_4_] was found to be associated with the EMIM cation, the conclusion can be made that the cation is actually limiting the intrinsic anodic potential of [EMIM][BF_4_]. The same conclusion was reached by Fogarty et al. in their liquid-phase study of [OMIM][BF_4_].^24^ Ong et al. also found that in [BMIM][BF_4_], [BMIM][PF_6_], and [P_13_][PF_6_], the HOMO is dominated by the cation states.^12^

These observations challenge the prevailing assumption that it is always the anion, which determines the oxidative stability.^12^

### 3.3 TFSI Anion-Based ILs

The newly measured ILs with the TFSI anion [EMMIM][TFSI], [P_4441_][TFSI], and [S_222_][TFSI] have similar UPS spectra. Their spectra are also similar to the UPS spectra of other TFSI anion-based ILs like [PYR_14_][TFSI], [DEME][TFSI], [EMIM][TFSI], [BMIM][TFSI], etc. This is because their spectra are mostly dominated by the anion. However, the low-energy region around 9–12 eV is still somewhat different in all of the TFSI anion- based ILs. As mentioned above, the low-binding-energy cutoff value of the TFSI anion-based IL vapors is about 8.6–8.7 eV.

The TFSI anion-based ILs are also the most thermally stable (see Table 1) under our experimental setup, i.e., they can be evaporated in high vacuum with minor thermal degradation.

Yoshimura et al. have published the liquid-phase [BMIM][TFSI] UPS spectrum, which coincides well with our gas-phase UPS spectra of TFSI-based ILs.^36^ They had the top of valence band at about 8.0 eV.

Strasser et al. also found that liquid- and vapor-phase [EMIM][TFSI] spectra are highly similar, with a shift of only about 0.7 eV.^20^

The M06 calculation is able to reproduce these spectra quite well. However, it tends to overestimate the intensity of the peak at a binding energy of 19.5 eV. It mostly yields HOMO and LUMO binding energies of 7.7 and 2.0 eV, respectively. Therefore, most TFSI anion-based IL ion pairs have 5.6–6.3 eV calculated gaps. Using UPS and IPS, Kanai et al. showed that [BMIM][TFSI] has a gap of 8.3 eV.^45^

The [P_4441_][TFSI] ion pair is structurally the largest of the ILs studied in this work. The calculation of this ion pair is also the most challenging due to the large number of conformers it has. The DOS depends significantly on the underlying ion-pair structure. Over 60 different [P_4441_][TFSI] conformers were evaluated at the M06/6-311++G** level of theory, which is very time-consuming.

It is important to point out a mistake in our recent paper about the TFSI anion-based ILs. Clearly, the DFT calculation of [PYR_14_][TFSI] has been performed on the nonsaturated pyrrole-based cation not on the saturated pyrrolidinium, which was measured experimentally.^19^ The PYR_14_ cation does not have an aromatic ring. However, since the [PYR_14_][TFSI] UPS spectrum is dominated by the TFSI anion, the simulated DOS does not change too much with the addition of hydrogens. However, the correct M06 [PYR_14_][TFSI] ion-pair energy gap is actually 5.78 eV not 4.88 eV.

Although [EMMIM][TFSI], [P_4441_][TFSI], and [S_222_][TFSI] have similar HOMO values, it is localized on different ions. The M06 calculation shows that in [PYR_14_, DEME, S_222_, P_4441_][TFSI], the HOMO is due to the TFSI anion, while in [EMIM][TFSI], and [EMMIM][TFSI], it is mixed. Using the MP2 calculation method, Kazemiabnavi et al. also found that the HOMO of [EMIM-HMIM][TFSI] is distributed all over the molecule.^3^ Fogarty et al. also identified both the cation and anion as the identity of the HOMO state in [BMIM][TFSI] and [OMIM][TFSI].^24^

Thus, the anodic limit in [EMIM][TFSI] and [EMMIM][TFSI] could be limited by the cation. Ong et al. also predicted that EMIM, BMIM, and HMIM cations are limiting the anodic potential when used in conjunction with PF_6_ and FAP anions. This observation challenges the prevailing assumption that it is always the anion that determines the oxidative stability.^12^

### 3.4 DCA and TCM Anions

The DFT calculation predicts that the HOMO of these DCA and TCM anion-based ILs is due to the π-orbitals of the anion. Furthermore, the top six molecular orbitals of [PYR_14_][DCA] and [PYR_14_][TCM] are associated with the π-orbitals of the anion.

Therefore, the DCA and TCM anions are (heavily) expected to limit the anodic limit of these ILs. Fogarty et al. also identified the anion (N 2p) as the identity of the HOMO state in [BMIM][DCA] and [OMIM][TCM].^24^ Furthermore, using resonant Auger spectroscopy, they were able to show that nitrogen is significantly contributing to the lowest-binding-energy feature.

The [EMIM][DCA] UPS spectrum is somewhat similar to the spectra of simple aromatic compounds such as benzylazide and methyl benzyl azides.^28^ The [EMIM][DCA] photoelectron spectrum also resembles the pure pyridine and methyl pyridine spectra,^27^ as expected. For example, the relatively sharp rise in intensity around 12 eV is very similar to pyridine. The double peak around 10 eV is also similar to pyridines, and it seems to be due to the aromatic nature of the compounds—similar features exist in the unsaturated cyclopentene, cyclohexene, and cyanobenzene, but not in the saturated cyclopentane and cyclohexane. Therefore, these features can be assigned to the π-orbitals of the cation.

The photoelectron spectra of [PYR_14_][TCM] and [PYR_14_][DCA] (see the Supporting Information) are very similar. Only small differences exist in the energies around 13 and 20.5 eV. Their UPS spectra are somewhat similar to the saturated hydrocarbon cyclopentane, which also has a distinct feature at a binding energy of 16 eV.^27^ Furthermore, all of the atomic rings composed of five atoms like cyclopentane, cyclopentene, and pyridines have a distinct 16 eV feature (about 1 eV width), which is similar to the [PYR_14_][DCA, TCM] 16.4 eV feature. The next cyclopentane feature at 19 eV also coincides with [PYR_14_][DCA].

However, the lowest-binding-energy feature at 8 eV of [EMIM or PYR_14_][DCA] is missing in all of these analogues. This again validates the claim that it is due to the DCA anion.

The DFT calculation is able to describe the electronic structure of [PYR_14_][TCM] very well (see Figure 1).

The [EMIM][DCA] double peak (HOMO) around 8.5 eV is also captured quite well, but the next double peak around 10.5 eV is shifted to higher energies by the M06 functional. It can also be seen that the hybrid DFT has some other inaccuracies in the description of the [EMIM][DCA] UPS spectrum.

For comparison, we also ran an MP2 calculation for [EMIM][DCA]. The MP2 calculation offers a better description of the [EMIM][DCA] spectrum, the peaks align better, and the intensities are also closer to the experiment (not shown). However, the MP2-calculated DOS needs about a 1 eV different shift compared to the other ILs. The electronic relaxation during the photoemission process could be the reason why both the M06 and MP2 calculations have difficulties in describing the electronic structure of [EMIM][DCA]. A similar effect has already been demonstrated in [EMIM][BF_4_].^21^ However, this problem will be pursued in a future study.

It should also be kept in mind that the experimental [EMIM][DCA] spectrum is the lowest-quality UPS spectra presented in this study. Thermal degradation products could also be present. This is due to the rather high volatility of this IL, which makes vapor-phase studies rather difficult.

## 4 Discussion

Asha et al. claimed that “the HOMO is always located in anions and the LUMO is mainly contributed by cations irrespective of the anions”.^8^ Very recently, they further claimed that the EW is solely decided by the HOMO energy of the pairing anions.^4^ This implies that eq 2 is always valid, i.e., the ion pair can be approximated by its constituent ions.

While this seems to hold for most of ILs, in the case of the strong anions like BF_4_^–^ and PF_6_^–^, the HOMO of the cation is actually at a higher energy; thus, the HOMO of the ion pair may also be determined by the cation. Based on eq 3, Roohi et al. pointed out that if the HOMO energy of the anion is larger than that of the cation, then the anodic stability is determined by the anion.^46^ In the case of the very negative HOMOs of fluorine-containing anions like BF_4_, PF_6_, and TFSI, the anodic stability of the IL could effectively be controlled by the cation.^46^ Furthermore, Roohi et al. also made the logical conclusion that the EWs of these fluorine-containing ILs can be equal, if the cation is the same. Fogarty et al. pointed out that the fact that the cation HOMO could also be the ion-pair HOMO might be surprising to some researchers.^24^ Tian et al. also pointed out that the anodic limit might be determined by oxidation of cations.^15^ Therefore, some further discussion about the localization of the HOMO and energy gaps is warranted.

The HOMO of [EMIM][OTF] is mostly localized on the OTF anion, but small contributions from the cation also exist. The HOMO of [PYR_14_][OTF] is due to the anion, more specifically, the oxygen atoms (possibly its lone pair electrons). The same conclusion was also reached by Fogarty et al. in their recent liquid-phase work.^24^ Using X-ray emission spectroscopy, Kanai et al. were also able to show experimentally that the oxygens are heavily contributing to the HOMO.^45^ They also showed that the energy gap of [BMIM][OTF] is 8.1 eV,^45^ which is in excellent agreement with our experimental [EMIM][OTF] HOMO energy of 8.4 eV. Therefore, in [EMIM][OTF], the cation may also influence the anodic stability, while in [PYR_14_][OTF], only the anion contributes to the HOMO.

A similar situation may exist in many other ILs. For example, in [S_222_][TFSI], the M06-calculated HOMO is localized only on the anion, while in [EMMIM][TFSI], it is distributed all over. Again, the cation may influence the localization of the HOMO orbital.

Therefore, we predict the anodic stability of [EMIM][OTF], [EMIM][TFSI], [EMMIM][TFSI], and [EMIM][BF_4_] to be (partly) limited by the cation. In most other cases, the anodic stability is limited by the anion, and in some IL ion pairs, the HOMO is distributed all over the molecule.

### 4.1 Electrochemical Stability Windows

As mentioned before, there are numerous theoretical studies that try to estimate the EWs of ILs. Most of them are based on the ion-pair approximation. It is impossible to give an overview of all of them, but to illustrate how many different methods, approaches, and results are published, some comparisons should be made.

Zhang et al. predicted the EW of [BMIM][PF_6_], [EMIM][TFSI], [EMIM][B(CN)_4_], [BMIM][BF_4_], and [BMIM][TFSI] 4.2–4.7.^47^ Thus, they predict a very small difference between the EWs of these ILs. This seems to be in contrast with experimental EWs and our gas-phase data and could be due to the use of a nonhybrid PBE functional in their calculations.

Using the thermodynamic method, Kazemiabnavi et al. calculated the EW of [C*_n_*MIM][OTF] to be about 0.5 V wider than [C*_n_*MIM][TFSI]. They claimed that the strength of the anions against oxidation is increasing in the following order: TFSI < OTF < BF_4_ < PF_6_.^3^^3^

However, Asha et al. put the TFSI anion as more stable than the OTF anion and ordered the anions this way: DCA < TFA < OTF < TFSI < BF_4_ < PF_6_.^4,8^ This in agreement with our data as generally the HOMO energies are increasing with increasing strength of the anion: DCA, OTF, TFSI, FSI, BF_4_, PF_6_. Therefore, we predict that the TFSI anion should be more stable than the OTF anion. Fogarty et al. also found that the TFSI anion-based ILs had larger HOMO binding energies (by about 0.1–0.7 eV) than the ILs with the OTF anion.^24^

By calculating isolated anions, Kazemiabnavi et al. claimed that the HOMO energy of TFSI anion is higher than the OTF anion, therefore making the TFSI anion less stable against oxidation than the OTF anion. This is an example of the failure of the isolated ion HOMO/LUMO methods (eqs 2 and 3), and it illustrates that the calculation of the cation and anion separately is not a good approximation to the ion pair. The TFSI anion is relatively large with a high degree of structural mutation and should not be modeled alone.

Another indication that the EW of the [C*_n_*MIM][TFSI] ILs may be off in the study by Kazemiabnavi et al. is the fact that the EW of the [C*_n_*MIM][TFSI] ILs calculated using the thermodynamic cycle method is underestimated, while the EWs of all of the other ILs are somewhat overestimated (vs the experimental EW). This discrepancy can be traced back to the predicted LUMO energies. Kazemiabnavi et al. show about a 0.6 eV difference in the (free) anion LUMO energies, while our M06 calculation shows that the LUMOs of [EMIM][OTF] and [EMIM][TFSI] are both localized on the cation and have almost equal energy. Therefore, not only the HOMO energies but also the LUMO energies of free anions may not represent the IL ion pair.

**Figure 2:**
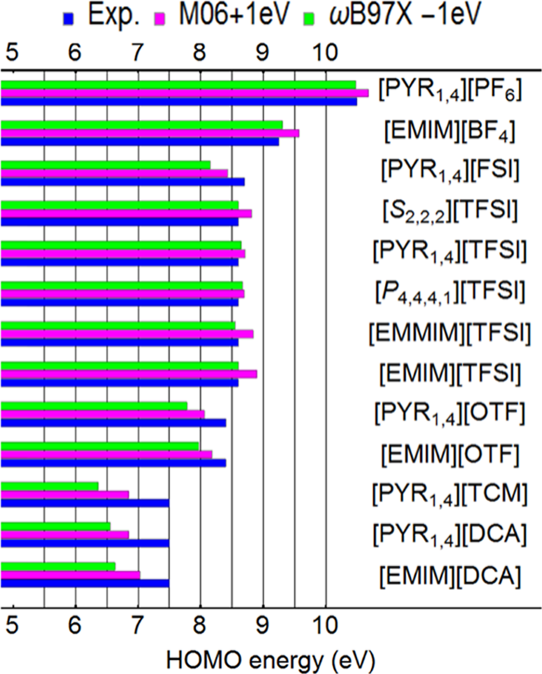
Graphical representation of the experimental HOMO energies compared to the adjusted M06 and wb97X-D calculated values.

In their recent study, Asha et al. estimated the EWs of [PYR_14_][PF_6_] as 6.54 V, [PYR_14_][TFSI] as 5.31 V, and [PYR_14_][OTF] as 4.62 V^8^ using the M06-L HOMO–LUMO method (eq 1). They also estimated the EWs of [PYR_14_][FSI] as 5.31 V, [PYR_14_][TFSI] as 5.35 V, [PYR_14_][OTF] as 4.62 V, and [PYR_14_][DCA] as 4.42 V using the same M06-L HOMO–LUMO method.^4^ Using the MP2 method, the corresponding values were 6.39, 6.35, 5.25, and 4.36 V.^4^ Finally, 5.38, 5.31, 4.93, and 4.39 V EWs were calculated using the thermodynamic cycle method.^4^ The order of the anions is in agreement with our experimental ion-pair measurements, i.e., [PYR_14_][FSI] has the largest EW, followed by [PYR_14_][TFSI], [PYR_14_][OTF], and [PYR_14_][DCA] (Figure 2).

The M06-L calculation of Asha et al. seems to be generally in agreement with our experimental HOMO differences, but systematically wrong on [PYR_14_][DCA]. Their [PYR_14_][DCA] HOMO–LUMO gap is probably overestimated, as their [PYR_14_][DCA] EW of 4.42 V is almost equal to their [PYR_14_][OTF] EW of 4.62 V. However, their [PYR_14_][OTF] EW could also be somewhat underestimated. The differences between the EWs of the ILs calculated using the thermodynamic cycle method are relatively small. For example, the predicted difference between the EWs of [PYR_14_][OTF] and [PYR_14_][DCA] is only 0.54 V, the difference between [PYR_14_][FSI] and [PYR_14_][OTF] is 0.45 V, and [PYR_14_][FSI]-[PYR_14_][DCA] is 0.99 V. However, there would be a relatively good agreement between our vapor-phase HOMO values and the EWs calculated by the thermodynamic cycle method if the former were contracted by about 30%. This is understandable as the PCM solvation model used in the thermodynamic cycle method may lower the oxidation values.^6^

In their recent work, Lian et al. estimated the EW of [EMIM][PF_6_] as 4.5 V, [PYR_14_][TFSI] as 4.2 V, [EMIM][TFSI] as 3.6 V, [EMIM][BF_4_] as 3.6 V, and [S_222_][TFSI] as 3.2 V using the ωB97X-D cation–anion HOMO–LUMO overlap method (eq 3).^9^ These values are probably too conservative. The EWs of [EMIM][TFSI] and [EMIM][BF_4_] can only be equal when the cation is limiting the anodic potential. As was mentioned above, the M06 calculation and the gas-phase data seem to indicate that in [EMIM][BF_4_], the cation is indeed limiting the anodic potential, while in [EMIM][TFSI], it is also influenced by the anion. Third, the EW of [S_222_][TFSI] should be roughly equal to or higher than [EMIM][TFSI]. For example, Fogarty et al. show [S_222_][TFSI] with the most negative HOMO out of the 37 ILs they studied.^24^ It is also difficult to understand how these rather small EW values have been derived, as ωB97X-D tends to overestimate the HOMO energies by about 1 eV (see Table 1).

Next, a comparison with the experimentally measured EW values should be performed. Unfortunately, it is very difficult to make comparisons with the experimental EWs, as there is a wide range of reported EWs in the scientific literature. The variance is due to the large number of factors affecting the final value, such as sweep rate, current cutoff value, and electrode (Pt, GC, Au, W, Ta) used.^13^ The very commonly used glassy carbon (GC) electrodes also limit the EWs.

The experimental cathodic stabilities of some of the ILs studied here are indeed high. [N_4441_][TFSI] achieved a cathodic limit of −3.5 V vs Ag/Ag^+.^^48^ [PYR_14_][TFSI] had a cathodic limit of −3.50 V (vs Ag/Ag^+^).^49^ The [P_4441_][TFSI] ILs achieved −3.2 V vs (Fc/Fc^+^).^5^ Matsumoto et al. have also demonstrated high cathodic stabilities of about −3.4 V (vs Fc/Fc^+^) for [P_6111_][TFSI], [N_1113_][TFSI], and [N_5555_][TFSI], which are similar ILs to [DEME][TFSI] and [P_4441_][TFSI].^50^ The [P_14 666_][FAP] IL achieved a cathodic limit of −3.4 V vs (Fc/Fc^+^) on mercury electrodes.^51^ Moreno et al. found a cathodic limit of −3.76 V (vs Ag/Ag^+^) for [PYR_14_][OTF].^52^ Mousavi found that ammonium-based cations were all limited at −3.4 V (vs Ag^+^/Ag).^53^ The EWs of ILs composed of these stable cations/anions can also be high. For example, [PYR_14_][TFSI] has a reported EW of 6.55–6.56 V^49^ and [DEME][BF_4_] has the corresponding value of 8.43 V.^54^

The anodic limit on GC electrodes seems to be limited to about +2.5 (vs Ag^+^/Ag).^5,55^ [PYR_14_][TFSI] on gold was shown to have anodic and cathodic stabilities of more than ± 3.5 V (vs Ag/Ag^+^).^56^ [DEME][BF_4_] on platinum also showed limits larger than ± 3.5 V (vs Ag/Ag^+^).^57^

There are conflicting data about the stability of ILs based on the BF_4_ anion. In some studies, the TFSI anion seems to have a larger EW than the same cation paired with the BF_4_ anion.^55^ However, there are also studies where the BF_4_ anion shows higher anodic potential than the TFSI anion. [Butylpyridinium][BF_4_] on GC has a reported anodic potential limit of +3.65 (vs Ag^+^/Ag).^49^ Zhang et al. also demonstrated that the EW of [DEME][BF_4_] is larger by about 1.5 V than [DEME][TFSI] and [PYR_14_][TFSI].^54^ Sato et al. showed that on platinum electrodes, [DEME][BF_4_] has about 0.5 V higher anodic limit than [DEME][TFSI].^57^ They made the correct conclusion that BF_4_ is more difficult to oxidize than TFSI. Thus, BF_4_ would seem to be at least 0.5 V more stable than TFSI. Indeed, our HOMO level differences imply a 0.65 V difference.

ILs based on the DCA (and TCM) anion have smaller EWs than most other ILs.^58^ Yoshida et al. found that in the oxidation scan, the DCA anion-based ILs (including [PYR_12_][DCA]) were stable up to +1.3 V (vs Ag^+^/Ag), resulting in their electrochemical windows in the range of 3.4–3.7 V.^59^ Yuan et al. claimed that the EW of [BMIM][DCA] is 3.63 V.^58^ Hayyan et al. found an anodic limit of 1.67 V (vs Ag^+^/Ag) and an EW of 4.62 V for [PYR_14_][DCA].^60^ This anodic limit is smaller than [PYR_14_][TFSI] by about 1.7 V, which is in good agreement with our gas-phase HOMO-level differences.

There is a correlation between the experimental EW, anodic limit, and the gas-phase HOMO energy. In other words, a more negative HOMO energy implies greater anodic stability and a larger EW. However, there is a wide distribution of EW values in the literature and more study on this is needed.

## 5 Conclusions

Gas-phase ion pairs of several ILs were investigated using valence band photoemission. The DFT calculation using the M06 functional was able to reproduce most of the spectral features, and it performs surprisingly well for most IL vapors (see Figure 1). The ωB97X-D functional offered a very similar level of performance at a somewhat higher computational cost. Systematic shifts were needed to bring the calculated DOS into agreement with the experimental UPS spectra. These shifts can probably be corrected with better functionals or better approaches (GW^43^).

In some cases ([PYR_14_][FSI], [PYR_14_][TCM]), the excellent agreement between the experimental UPS spectrum and the calculated DOS validates the conformer found for the IL ion pair and provides indirect experimental evidence for the structure of ionic liquid vapors. This is due to the sensitivity of the UPS spectrum on the underlying ion-pair structure.

The prevalent assumption that the cation sets the cathodic limit and the anion sets the anodic limit may not be valid for some ILs.^12^ When a “weak” cation is paired with a “strong” anion ([EMIM][BF_4_], for example), the cation can determine the oxidative stability.^50^ However, the approximation still holds in most cases.

It can be shown that many recent theoretical estimations of IL EWs are not accurate. This is not only due to the untested validity of the ion-pair approximation used in the calculation. It seems that a calculation method that incorporates true Hartree–Fock exchange (or the GW approach) is a must for a correct ab initio description of IL ion pairs. The new UPS data will help to validate and further develop the numerous works on the electronic structure and electrochemical stability limits of ILs.

The new data also provide strong support to the argument that the modeling of IL cations and anions separately is incorrect and could lead to wrong conclusions.

It is very difficult to make comparisons with the experimental EWs, as there is a wide range of reported EWs in the scientific literature. However, in some cases, a good correlation between the new gas-phase data and the experimental EW is found.

There is no clear direct connection between the electronic gap and the electrochemical gap. The ion-pair approximation always overestimates the true EW,^3^ but is generally considered to provide an upper bound for the true stability potential window.^7^ It is also computationally less expensive than other methods. Peljo and Girault claim that these kinds of ion-pair approximations to the EW should be discarded completely.^61^ However, we will not go so far and believe that further study of the vapor- and liquid-phase electronic structure and also the excited (LUMO) states of the ion pairs of these ILs is necessary to make final conclusions.

At this point, still a limited number (10+) of experimental photoelectron spectra of IL vapors are published. The liquid IL energy gaps determined by the IPS/UPS method are in excellent agreement with our gas-phase HOMO data and the M06 (+1 eV) calculations. Indeed, the UPS/IPS method is a direct probe of the valence and conduction bands. Therefore, this method is recommended for future IL studies.

## Supporting Information Available

Raw “as-measured” UPS spectra and details about the ionic liquid samples (PDF)

## Notes

The raw data of this study are available from the corresponding author upon reasonable request.

## References

[ref1] Lian C.; Liu H.; Wu J. Ionic Liquid Mixture Expands the Potential Window and Capacitance of a Supercapacitor in Tandem. J. Phys. Chem. C 2018, 122, 18304–18310. 10.1021/acs.jpcc.8b05148.

[ref2] Jónsson E. Ionic liquids as electrolytes for energy storage applications—A modelling perspective. Energy Storage Mater. 2020, 25, 827–835. 10.1016/j.ensm.2019.08.030.

[ref3] Kazemiabnavi S.; Zhang Z.; Thornton K.; Banerjee S. Electrochemical Stability Window of Imidazolium-Based Ionic Liquids as Electrolytes for Lithium Batteries. J. Phys. Chem. B 2016, 120, 5691–5702. 10.1021/acs.jpcb.6b03433. 27266487

[ref4] Asha S.; Vijayalakshmi K. P.; George B. K. Pyrrolidinium-based ionic liquids as electrolytes for lithium batteries: A Computational Study. Int. J. Quantum Chem. 2019, e26014 10.1002/qua.26014.

[ref5] Xue Z.; Qin L.; Jiang J.; Mu T. G. Thermal, electrochemical and radiolytic stabilities of ionic liquids. Phys. Chem. Chem. Phys. 2018, 20, 8382–8402. 10.1039/C7CP07483B. 29503990

[ref6] Borodin O.; Behl W.; Jow T. R. Oxidative Stability and Initial Decomposition Reactions of Carbonate, Sulfone, and Alkyl Phosphate-Based Electrolytes. J. Phys. Chem. C 2013, 117, 8661–8682. 10.1021/jp400527c.

[ref7] Binninger T.; Marcolongo A.; Mottet M.; Weber V. Comparison of computational methods for the electrochemical stability window of solid-state electrolyte materials. J. Mater. Chem. A 2020, 8, 1347–1359. 10.1039/C9TA09401F.

[ref8] Asha S.; Vijayalakshmi K. P.; George B. K. Electronic structural studies of pyrrolidinium-based ionic liquids for electrochemical application. Int. J. Quantum Chem. 2019, 119, e25972 10.1002/qua.25972.

[ref9] Lian C.; Liu H.; Li C.; Wu J. Hunting Ionic Liquids with Large Electrochemical Potential Windows. AIChE J. 2019, 65, 804–810. 10.1002/aic.16467.

[ref10] Ilawe N.; Fu J.; Ramanathan S.; Wong B.; Wu J. Chemical and Radiation Stability of Ionic Liquids: A Computational Screening Study. J. Phys. Chem. C 2016, 120, 27757–27767. 10.1021/acs.jpcc.6b08138.

[ref11] Haskins J. B.; Bauschlicher C. W.; Lawson J. W. Ab Initio Simulations and Electronic Structure of Lithium-Doped Ionic Liquids: Structure, Transport, and Electrochemical Stability. J. Phys. Chem. B 2015, 119, 14705–14719. 10.1021/acs.jpcb.5b06951. 26505208

[ref12] Ong S. P.; Andreussi O.; Wu Y.; Marzari N.; Ceder G. Electrochemical Windows of Room-Temperature Ionic Liquids from Molecular Dynamics and Density Functional Theory Calculations. Chem. Mater. 2011, 23, 2979–2986. 10.1021/cm200679y.

[ref13] Jónsson E.; Johansson P. Electrochemical oxidation stability of anions for modern battery electrolytes: a CBS and DFT study. Phys. Chem. Chem. Phys. 2015, 17, 3697–3703. 10.1039/C4CP04592K. 25557392

[ref14] Zhang Y.; Shi C.; Brennecke J. F.; Maginn E. J. Refined Method for Predicting Electrochemical Windows of Ionic Liquids and Experimental Validation Studies. J. Phys. Chem. B 2014, 118, 6250–6255. 10.1021/jp5034257. 24823869

[ref15] Tian Y.-H.; Goff G. S.; Runde W. H.; Batista E. R. Exploring Electrochemical Windows of Room-Temperature Ionic Liquids: A Computational Study. J. Phys. Chem. B 2012, 116, 11943–11952. 10.1021/jp303915c. 22946441

[ref16] Ikari T.; Keppler A.; Reinmöller M.; Beenken W. J. D.; Krischok S.; Marschewski M.; Maus-Friedrichs W.; Höfft O.; Endres F. Surface Electronic Structure of Imidazolium-Based Ionic Liquids Studied by Electron Spectroscopy. e-J. Surf. Sci. Nanotechnol. 2010, 8, 241–245. 10.1380/ejssnt.2010.241.

[ref17] Höfft O.; Bahr S.; Himmerlich M.; Krischok S.; Schaefer J.; Kempter V. Electronic structure of the surface of the ionic liquid [EMIM][Tf2N] studied by metastable Impact Electron Spectroscopy (MIES), UPS, and XPS. Langmuir 2006, 22, 7120–7123. 10.1021/la060943v. 16893200

[ref18] Strasser D.; Goulay F.; Belau L.; Kostko O.; Koh C.; Chambreau S. D. Tunable Wavelength Soft Photoionization of Ionic Liquid Vapors. J. Phys. Chem. A 2010, 114, 879–883. 10.1021/jp909727f. 19957958

[ref19] Kuusik I.; Kook M.; Pärna R.; Kivimäki A.; Käämbre T.; Reisberg L.; Kikas A.; Kisand V. The electronic structure of ionic liquids based on the TFSI anion: A gas phase UPS and DFT study. J. Mol. Liq. 2019, 294, 111580 10.1016/j.molliq.2019.111580.

[ref20] Strasser D.; Goulay F.; Kelkar M. S.; Maginn E. J.; Leone S. R. Photoelectron Spectrum of Isolated Ion-Pairs in Ionic Liquid Vapor. J. Phys. Chem. A 2007, 111, 3191–3195. 10.1021/jp071323l. 17411023

[ref21] Kuusik I.; Tarkanovskaja M.; Kruusma J.; Kisand V.; Tõnisoo A.; Lust E.; Nõmmiste E. Valence band photoelectron spectra of [EMIM][BF4] ionic liquid vapor: Evidences of electronic relaxation. J. Mol. Liq. 2016, 223, 939–942. 10.1016/j.molliq.2016.08.114.

[ref22] Kuusik I.; Berholts M.; Kruusma J.; Kisand V.; Tõnisoo A.; Lust E.; Nõmmiste E. Valence electronic structure of [EMIM][BF4] ionic liquid: photoemission and DFT+D study. RSC Adv. 2018, 8, 30298–30304. 10.1039/C8RA05865B. PMC9085424 35546846

[ref23] Kuusik I.; Berholts M.; Kruusma J.; Tõnisoo A.; Lust E.; Nommiste E.; Kisand V. Valence electronic structure of [EMIM][B(CN)4]: ion-pair vs bulk description. RSC Adv. 2019, 9, 33140 10.1039/C9RA06762K. PMC9073151 35529163

[ref24] Fogarty R. M.; Palgrave R. G.; Bourne R. A.; Handrup K.; Villar-Garcia I. J.; Payne D. J.; Hunt P. A.; Lovelock K. R. J. Electron spectroscopy of ionic liquids: experimental identification of atomic orbital contributions to valence electronic structure. Phys. Chem. Chem. Phys. 2019, 21, 18893 10.1039/C9CP02200G. 31441923

[ref25] Pärna R.; Sankari R.; Kukk E.; Nõmmiste E.; Valden M.; Lastusaari M.; Kooser K.; Kokko K.; Hirsimäki M.; Urpelainen S. P.; Kivimäki A.; Pankratov V.; Reisberg L.; Hennies F.; Tarawneh H.; Nyholm R.; Huttula M. FinEstBeaMS—A wide-range Finnish-Estonian Beamline for Materials Science at the 1.5 GeV storage ring at the MAX IV Laboratory. Nucl. Instrum. Methods Phys. Res., Sect. A 2017, 859, 83–89. 10.1016/j.nima.2017.04.002.

[ref26] Yencha A. J.; Cormack A. J.; Donovan R. J.; Hopkirk A.; King G. C. Threshold photoelectron spectroscopy of HF and DF in the outer valence ionization region. J. Phys. B: At., Mol. Opt. Phys. 1999, 32, 2539–2550. 10.1088/0953-4075/32/11/306.

[ref27] Kimura K. Handbook of HeI Photoelectron Spectra of Fundamental Organic Molecules: Ionization Energies, Ab Initio Assignments, and Valence Electronic Structure for 200 Molecules; Japan Scientific Societies Press: Tokyo, 1981.

[ref28] Pinto R. M. V.-d.-R. Photoelectron Spectroscopy of Nitrogen Containing Molecules of Biological and Industrial Interest; University of Lisbon: Lisbon, 2011.

[ref29] Fogarty R. M.; Matthews R. P.; Ashworth C. R.; Brandt-Talbot A.; Palgrave R. G.; Bourne R. A.; Hoogerstraete T. V.; Hunt P. A.; Lovelock K. R. J. Experimental validation of calculated atomic charges in ionic liquids. J. Chem. Phys. 2018, 148, 193817 10.1063/1.5011662. 30307226

[ref30] Lage-Estebanez I.; del Olmo L.; Lopez R.; de la Vega J. M. G. The Role of Errors Related to DFT Methods in Calculations Involving Ion Pairs of Ionic Liquids. J. Comput. Chem. 2017, 38, 530–540. 10.1002/jcc.24707. 28133839

[ref31] Reinmöller M.; Ulbrich A.; Ikari T.; Preis J.; Höfft O.; Endres F.; Krischok S.; Beenken W. J. D. Theoretical reconstruction and element wise analysis of photoelectron. Phys. Chem. Chem. Phys. 2011, 13, 19526–19533. 10.1039/c1cp22152c. 21971301

[ref32] Krischok S.; Eremtchenko M.; Himmerlich M.; Lorenz P.; Uhlig J.; Neumann A.; Öttking R.; Beenken W. J. D.; Himmerlich M.; Lorenz P.; Höfft O.; Bahr S.; Kempter V.; Schaefer J. A. A Comparative Study on the Electronic Structure of the 1-Ethyl-3-Methylimidazolium Bis(trifluoromethylsulfonyl)amide RT-Ionic Liquid by Electron Spectroscopy and First Principles Calculations. Z. Phys. Chem. 2006, 220, 1407–1416. 10.1524/zpch.2006.220.10.1407.

[ref33] Kurisaki T.; Tanaka D.; Inoue Y.; Wakita H.; Minofar B.; Fukuda S.; Ishiguro S.; Umebayashi Y. Surface Analysis of Ionic Liquids with and without Lithium Salt Using X-ray Photoelectron Spectroscopy. J. Phys. Chem. B 2012, 116, 10870–10875. 10.1021/jp301658k. 22853737

[ref34] Nishi T.; Iwahashi T.; Yamane H.; Ouchi Y.; Kanai K. K. Electronic structures of ionic liquids Cnmim BF4 studied by ultraviolet photoemission, inverse photoemission, and near-edge X-ray absorption fine structure spectroscopies. Chem. Phys. Lett. 2008, 455, 213–217. 10.1016/j.cplett.2008.01.049.

[ref35] Ulbrich A.; Reinmöller M.; Beenken W. J. D.; Krischok S. Photoelectron spectroscopy on ionic liquid surfaces—Theory and experiment. J. Mol. Liq. 2014, 192, 77–86. 10.1016/j.molliq.2014.01.007.

[ref36] Yoshimura D.; Yokoyama T.; Nishi T.; Ishii H.; Ozawa R.; Hamaguchi H.; Seki K. Electronic structure of ionic liquids at the surface studied by UV photoemission. J. Electron Spectrosc. Relat. Phenom. 2005, 144–147, 319–322. 10.1016/j.elspec.2005.01.181.

[ref37] Kanai K.; Nishi T.; Iwahashi T.; Ouchi Y.; Seki K.; Harada Y.; Shin S. Anomalous electronic structure of ionic liquids determined by soft x-ray emission spectroscopy: Contributions from the cations and anions to the occupied electronic structure. J. Chem. Phys. 2008, 129, 224507 10.1063/1.3036925. 19071928

[ref38] Mardirossian N.; Head-Gordon M. Thirty years of density functional theory in computational chemistry: an overview and extensive assessment of 200 density functionals. Mol. Phys. 2017, 115, 2315–2372. 10.1080/00268976.2017.1333644.

[ref39] Fu Y.; Liu L.; Yu H. Z.; Wang Y. M.; Guo Q. X. Quantum-Chemical Predictions of Absolute Standard Redox Potentials of Diverse Organic Molecules and Free Radicals in Acetonitrile. J. Am. Chem. Soc. 2005, 127, 7227–7234. 10.1021/ja0421856. 15884964

[ref40] Nagy A.; Adachi H. Total energy versus one-electron energy differences in the excited-state density functional theory. J. Phys. B: At., Mol. Opt. Phys. 2000, 33, L585–L589. 10.1088/0953-4075/33/16/104.

[ref41] Yildirim H.; Haskins J. B.; Bauschlicher C. W.; Lawson J. W. Decomposition of Ionic Liquids at Lithium Interfaces. 1. Ab Initio Molecular Dynamics Simulations. J. Phys. Chem. C 2017, 121, 28214–28234. 10.1021/acs.jpcc.7b09657.

[ref42] Haskins J.; Yildirim H.; Bauschlicher C.; Lawson J. Decomposition of Ionic Liquids at Lithium Interfaces. 2. Gas Phase Computations. J. Phys. Chem. C 2017, 121, 28235–28248. 10.1021/acs.jpcc.7b09658.

[ref43] Kahk J. M.; Kuusik I.; Kisand V.; Lovelock K. R. J.; Lischner J. Frontier Orbitals and Quasiparticle Energy Levels in Ionic Liquids. npj Comput. Mater. 2020, 6, 792 10.1038/s41524-020-00413-4.

[ref44] Kuusik I.; Tarkanovskaja M.; Kruusma J.; Reedo V.; Välbe R.; Lõhmus A.; Kisand V.; Lust E.; Kukk E.; Nõmmiste E. Near threshold photodissociation study of EMIMBF4 vapor. RSC Adv. 2015, 5, 6834–6842. 10.1039/C4RA12775G.

[ref45] Kanai K.; Nishi T.; Iwahashi T.; Ouchi Y.; Seki K.; Harada Y.; Shin S. Electronic structures of imidazolium-based ionic liquids. J. Electron. Spectrosc. Relat. Phenom. 2009, 174, 110–115. 10.1016/j.elspec.2009.02.004.

[ref46] Roohi H.; Salehi R. Molecular engineering of the electronic, structural, and electrochemical properties of nanostructured 1-methyl-4-phenyl 1,2,4 triazolium-based [PhMTZ][X1–10] ionic liquids through anionic changing. Ionics 2018, 24, 483–504. 10.1007/s11581-017-2198-3.

[ref47] Zhang Q.; Han Y.; Wang Y.; Ye S.; Yan T. Comparing the differential capacitance of two ionic liquid electrolytes: Effects of specific adsorption. Electrochem. Commun. 2014, 38, 44–46. 10.1016/j.elecom.2013.10.027.

[ref48] Mousavi M.; Dittmer A.; Wilson B.; Hu J.; Stein A.; Bühlmann P. Unbiased quantification of the electrochemical stability limits of electrolytes and ionic liquids. J. Electrochem. Soc. 2015, 162, A2250–A2258. 10.1149/2.0271512jes.

[ref49] Li Q.; Jiang J.; Li G.; Zhao W.; Zhao X.; Mu T. The electrochemical stability of ionic liquids and deep eutectic solvents. Sci. China: Chem. 2016, 59, 571–577. 10.1007/s11426-016-5566-3.

[ref50] Matsumoto H.; Sakaebe H.; Tatsumi K. Preparation of room temperature ionic liquids based on aliphatic onium cations and asymmetric amide anions and their electrochemical properties as a lithium battery electrolyte. J. Power Sources 2005, 146, 45–50. 10.1016/j.jpowsour.2005.03.103.

[ref51] Rogers E. I.; Sljukic B.; Hardacre C.; Compton R. G. Electrochemistry in Room-Temperature Ionic Liquids: Potential Windows at Mercury Electrodes. J. Chem. Eng. Data 2009, 54, 2049–2053. 10.1021/je800898z.

[ref52] Moreno M.; Montanino M.; Carewska M.; Appetecchi G. B.; Jeremias S.; Passerini S. Water-soluble, triflate-based, pyrrolidinium ionic liquids. Electrochim. Acta 2013, 99, 108–116. 10.1016/j.electacta.2013.03.046.

[ref53] Mousavi M. P. S.; Kashefolgheta S.; Stein A.; Bühlmann P. Electrochemical Stability of Quaternary Ammonium Cations:An Experimental and Computational Study. J. Electrochem. Soc. 2016, 163, H74–H80. 10.1149/2.0671602jes.

[ref54] Zhang S.; Brahim S.; Maat S. High-voltage operation of binder-free CNT supercapacitors using ionic liquid electrolytes. J. Mater. Res. 2017, 33, 1179–1188.

[ref55] Mousavi M.; Wilson B.; Kashefolgheta S.; Anderson E. L.; He S.; Bühlmann P.; Stein A. Ionic Liquids as Electrolytes for Electrochemical Double-Layer Capacitors: Structures that Optimize Specific Energy. ACS Appl. Mater. Interfaces 2016, 8, 3396–3406. 10.1021/acsami.5b11353. 26771378

[ref56] Kokorin A. Ionic Liquids for the Future Electrochemical Applications. In Ionic Liquids: Applications and Perspectives; Bod Books on Demand, 2011.

[ref57] Sato T.; Marukane S.; Morinaga T. Ionic Liquids for the Electric Double Layer Capacitor Applications. In Applications of Ionic Liquids in Science and Technology; InTech, 2011; pp 109−134.

[ref58] Yuan W.-L.; Yang X.; He L.; Xue Y.; Qin S.; Tao G.-H. Viscosity, Conductivity, and Electrochemical Property of Dicyanamide Ionic Liquids. Front. Chem. 2018, 6, 59 10.3389/fchem.2018.00059. 29600245 PMC5862833

[ref59] Yoshida Y.; Baba O.; Saito G. Ionic Liquids Based on Dicyanamide Anion: Influence of Structural Variations in Cationic Structures on Ionic Conductivity. J. Phys. Chem. B 2007, 111, 4742–4749. 10.1021/jp067055t. 17474700

[ref60] Hayyan M.; Mjalli F. S.; Hashim M. A.; AlNashef I. M.; Mei T. X. Investigating the electrochemical windows of ionic liquids. J. Ind. Eng. Chem. 2013, 19, 106–112. 10.1016/j.jiec.2012.07.011.

[ref61] Peljo P.; Girault H. H. Electrochemical potential window of battery electrolytes: the HOMO–LUMO misconception. Energy Environ. Sci. 2018, 11, 2306 10.1039/C8EE01286E.

