# Supplementary material for: Ionic Liquid Vapors in Vacuum: Possibility to Derive Anodic Stabilities from DFT and UPS
Source: ACS Omega. 2021 Feb 15;6(8):5255–65. doi: 10.1021/acsomega.0c05369 (PMC7931180; doi:10.1021/acsomega.0c05369)
Supplement: Supplementary file 1 — ao0c05369_si_001.pdf [file ao0c05369_si_001.pdf]

Supporting Information.

Ionic liquid vapors in vacuum: possibility to derive anodic stabilities from DFT and UPS

Ivar Kuusik[a*], Mati Kook[a], Rainer Pärna[a], Vambola Kisand[a]

[a] Institute of Physics, University of Tartu, W. Ostwaldi 1, EE-50411 Tartu, Estonia

Abbreviation	Name	Supplier	Stated Purity	Evaporation temperature (°C)
[S_{222}][TFSI]	Diethylmethylsulfonium Bis(trifluoromethylsulfonyl)imide	Iolitec	99.9%	200-220
[P_{4441}][TFSI]	Tributylmethylphosphonium Bis(trifluoromethanesulfonyl)imide	TCI		200-220
[EMIM][TFSI]	1-Ethyl-3-methyl-imidazolium bis(trifluoromethylsulfonyl)imide	Sigma		250-280
[EMMIM][TFSI]	1-Ethyl-2-methyl-3-methyl-imidazolium Bis(trifluoromethylsulfonyl)imide	Iolitec	99.9%	220
[EMIM][OTF]	1-Ethyl-3-methyl-imidazolium Trifluoromethanesulfonate	Iolitec	99.2%	235
[PYR_{14}][OTF]	1-Butyl-1-methylpyrrolidinium Trifluoromethanesulfonate	TCI		230-250
[PYR_{14}][DCA]^{S}	1-Butyl-1-methylpyrrolidinium dicyanamide	TCI	99.9%	160-180
[EMIM][DCA]**	1-Ethyl-3-methyl-imidazolium dicyanamide	Iolitec	98.1%	140
[PYR_{14}][TCM]	1-Butyl-1-methylpyrrolidinium tricyanomethanide	Iolitec	98%	160
[PYR_{14}][TFSI]	1-Butyl-1-methylpyrrolidinium Bis(trifluoromethanesulfonyl)imide	TCI		200-220
[PYR_{14}][FSI]	1-Butyl-1-methylpyrrolidinium Bis(fluorosulfonyl)imide	TCI		185
[EMIM][BF_4]†	1-Ethyl-3-methyl-imidazolium Tetrafluoroborate	Sigma		190
[PYR_{14}][PF_6]^{S}	1-Butyl-1-methylpyrrolidinium Hexafluorophosphate	TCI		270

Table S1. Details about the the ILs investigated in this work.

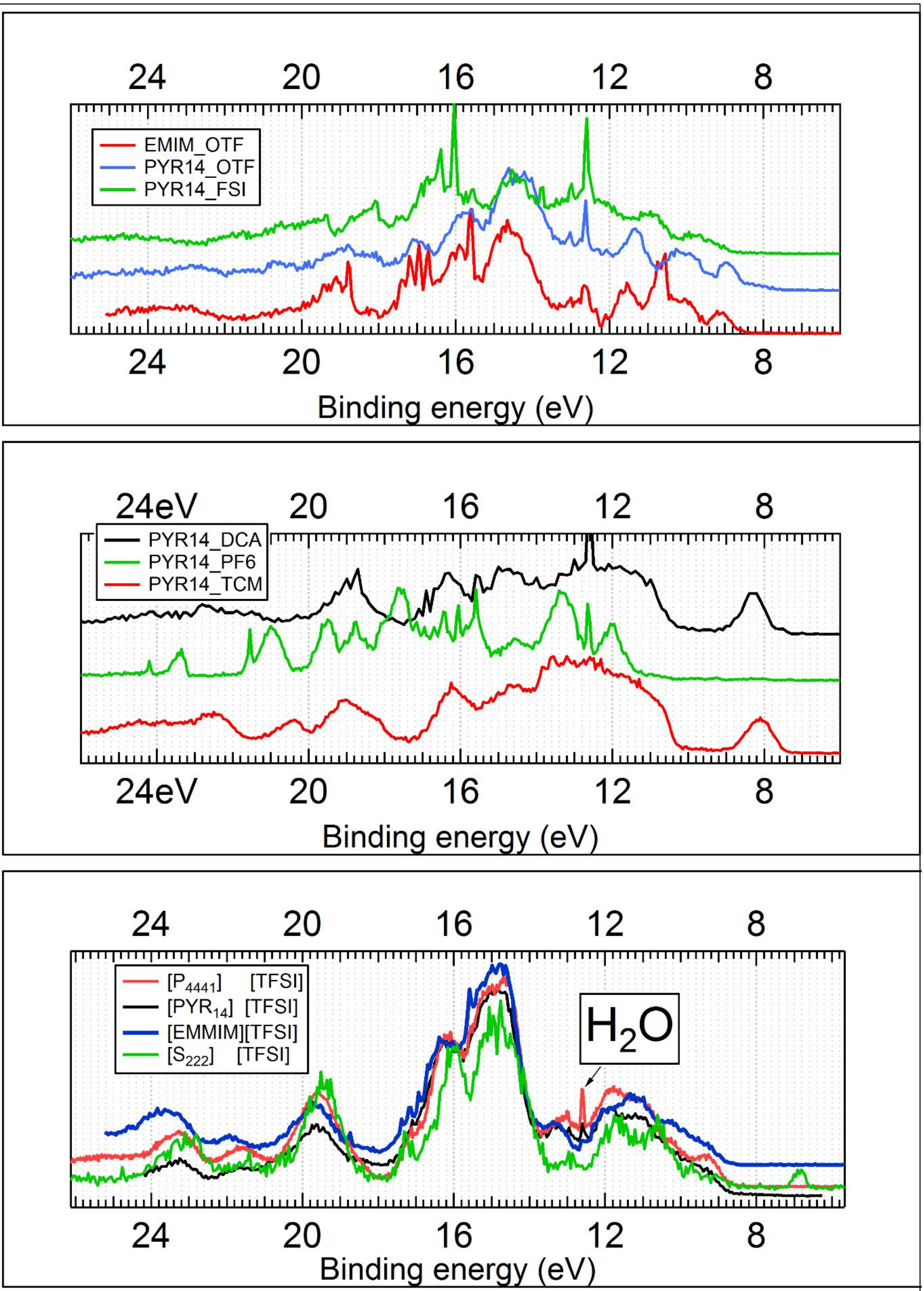

Figure S1. The raw UPS spectra of the ILs of this study.

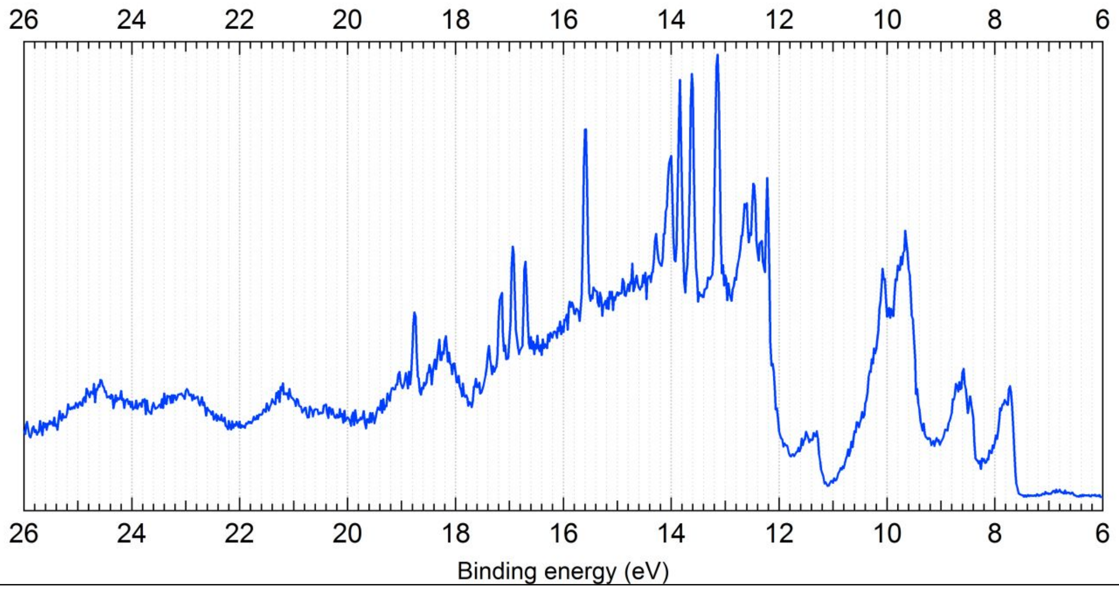

Figure S2. The raw UPS spectra of the [EMIM][DCA]. The spectrum has some contributions from contaminants (HCN) and rest gases (N_2, H_2O)
